# Multi-Objective Loss Function For Free Energy Calculations

**DOI:** 10.1145/3765612.3767254

**Published:** 2025-12-10

**Authors:** Libina Bovan Thomas, Ali Risheh, Negin Forouzesh

**Affiliations:** California State University, Los Angeles, Los Angeles, California, USA; University of California, Irvine, Irvine, California, USA; California State University, Los Angeles, Los Angeles, California, USA

**Keywords:** Drug discovery, Deep learning, Graph Convolutional Neural Networks, Binding Free Energy

## Abstract

Accurate estimation of binding free energy (ΔΔG) remains a critical challenge in drug discovery, directly influencing the development of effective therapeutics. Physics-based computational methods–such as molecular dynamics simulations and implicit solvent modeling–offer rigorous, atomistic insights into molecular recognition but are often constrained by computational costs and limited scalability. In contrast, deep learning models, particularly graph convolutional networks (GCNs), have demonstrated the ability to rapidly predict molecular properties by learning hierarchical representations from large-scale chemical data, yet frequently lack explicit incorporation of physical laws, leading to potential issues with interpretability and generalizability. Hybrid physics-guided neural networks seek to overcome these limitations by embedding physically meaningful features and constraints within deep learning architectures. In this study, we introduce a multi-objective loss function that simultaneously optimizes empirical error, structural similarity, and physical consistency by integrating molecular fingerprints with physics-based features such as electrostatic and van der Waals energies in a unified GCN framework. Applied to 65–75 host–guest systems, this approach yields substantial improvements in entropy (TΔS) prediction accuracy, reducing the mean absolute difference (MAD) from 5.80 to 1.07 kcal/mol, while also enhancing the accuracy and convergence of binding free energy (ΔΔG) predictions (MAD reduced to 1.54 kcal/mol), mitigating overfitting, and improving model transferability to larger complexes. These results demonstrate that principled loss function engineering is pivotal not only for accurate entropy estimation but also for enhancing the reliability and interpretability of binding free energy predictions in molecular machine learning models.

## 1 INTRODUCTION

Drug discovery is a fundamental process in combating diseases and improving patient health and life expectancy, yet it remains costly, time-consuming, and burdened by high failure rates [32]. Central to this process is the interaction between proteins and ligands, which determines the therapeutic efficacy of drug candidates. Accurate calculation of protein–ligand binding free energy has emerged as a critical tool in modern drug discovery, enabling quantitative prediction of binding affinities that guide hit selection and lead optimization [10]. Advances in computational techniques, such as physics-based free energy perturbation methods, have made it possible to reliably estimate these binding energies, providing valuable mechanistic insights into protein conformational changes and ligand interactions [2]. When combined with artificial intelligence and machine learning approaches, these calculations facilitate the efficient exploration of vast chemical spaces, improve prioritization of compounds, and reduce experimental costs and failures [5]. This integration of computational free energy methods with ML-driven strategies is transforming drug discovery from a largely empirical and resource-intensive endeavor into a predictive, data-driven science, accelerating the development of safer and more effective therapeutics [18].

**Figure 1:**
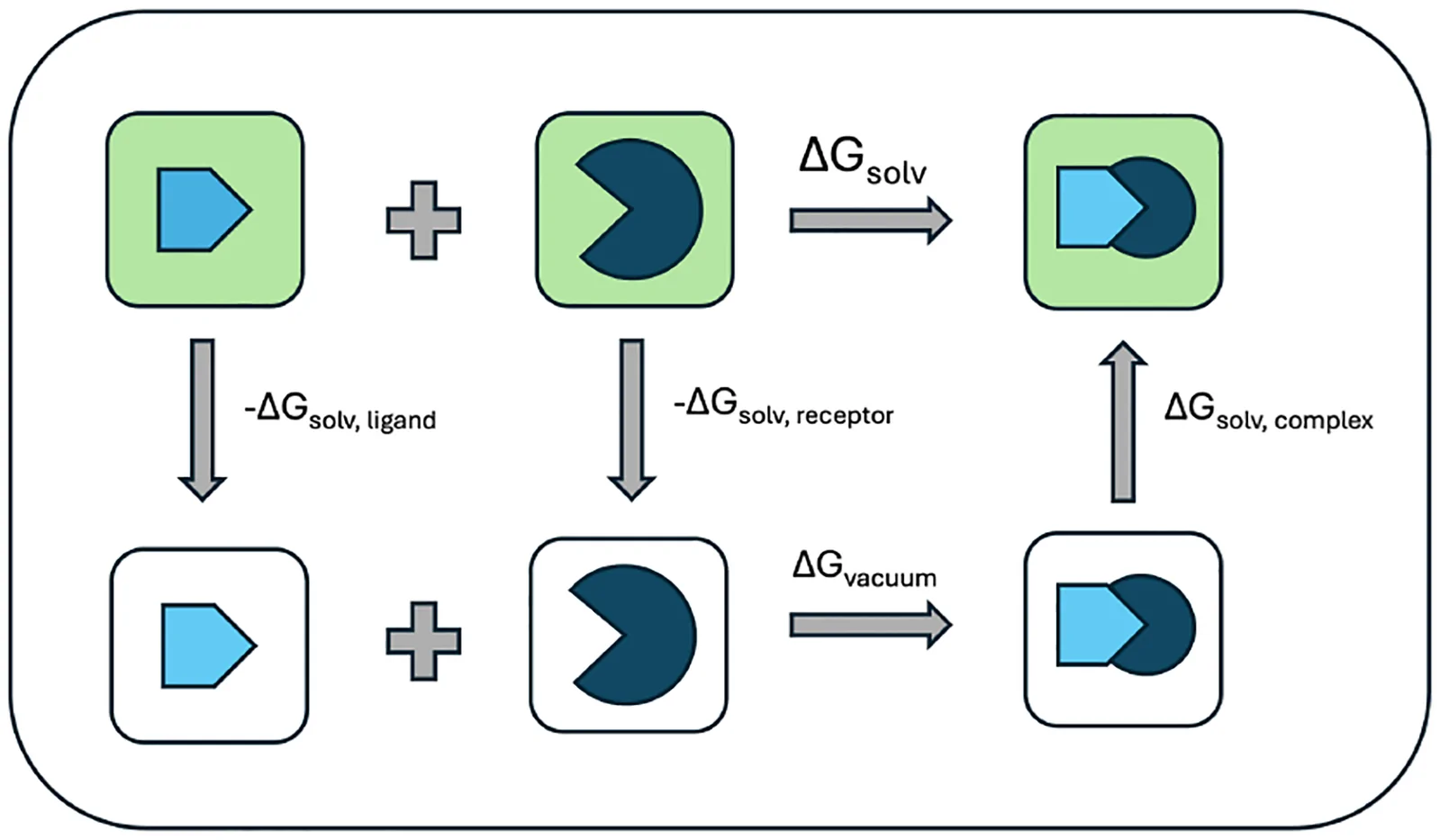
The thermodynamic cycle for ΔΔG calculation in implicit solvent modeling computes the binding free energy in aqueous environments as the difference between binding in vacuum and solvation of the complex, receptor, and ligand individually.

This paper aims to predict the binding free energy, which measures the thermodynamic favorability of a ligand binding to a biological target such as a protein or enzyme [20]. A more negative binding free energy typically indicates a stronger, more stable interaction, making ΔΔG a direct proxy for molecular affinity. Therefore, ΔΔG is a measure of the thermodynamic stability of a ligand-receptor complex and is determined by the difference in free energy between the complex and the individual binding components (ligand and receptor) [12]. Building on the growing importance of accurate binding free energy prediction in drug discovery, a wide spectrum of computational methods has been developed to estimate protein–ligand affinities, each balancing physical rigor and computational efficiency. At the most rigorous end, alchemical free energy methods such as free energy perturbation (FEP) and thermodynamic integration use molecular dynamics (MD) simulations to provide highly accurate estimates of binding free energies by explicitly modeling the process of ligand binding and unbinding, though these approaches are often computationally intensive and complex to implement [9, 1]. Such complexities increase the importance of advanced computational methods that can simulate and predict these interactions. The rise of computer-aided drug design has transformed the pharmaceutical discovery process, providing innovative tools and approaches that accelerate drug development, lower costs, and reduce risks [27]. Implicit solvent modeling is one such popular computational method for estimating binding free energy based on a thermodynamic cycle shown in Figure 1, which breaks down the binding process into its constituent parts [15]. This approach accounts for different energetic contributions, including solvation and desolvation energies, as well as vacuum terms that describe the internal interactions within the complex. However, traditional implicit solvent methods, such as Poisson-Boltzmann and Generalized Born, often depend on approximations that may not scale well with larger or flexible systems [24]. The main objective of this paper is to create a more accurate and efficient pipeline for predicting binding free energy while ensuring the results remain consistent with physics-based simulations. Recent advances in physics-informed machine learning (PIML) have demonstrated that embedding domain-specific physical knowledge into neural networks can significantly improve both predictive accuracy and model interpretability, especially in scientific domains where data are scarce or noisy and physical laws are well-established [25]. The initial implementation of Physics-Guided Neural Networks (PGNN) [11] demonstrated how that goal was achievable. Building on this concept, a Physics-Guided Rule-based Graph Convolutional Network (PG-RGCN) was developed to predict binding entropy [31]. That work also involved integrating Normal Mode Analysis (NMA) simulations to generate entropy data for various host-guest systems. These simulations provided the vibrational, rotational, and translational entropy values for the host, guest, and complex [3].

Although PIML frameworks have shown promise in scientific domains with well-established physical laws, their application to binding free energy prediction is not straightforward. Each term in the binding free energy equation–such as enthalpic and entropic contributions, solvation/desolvation energies–had to be clearly defined and appropriately encoded within the model to ensure both accuracy and physical consistency. This is particularly important because, in the context of drug discovery, even small errors in predicted binding affinities can lead to costly experimental failures. The PG-RGCN model, as originally developed [31], focused on minimizing Root Mean Square Error (RMSE). As the research progressed, it became clear that achieving better accuracy required moving beyond traditional error minimization because this limitation often led to overfitting and reduced generalization performance. While reducing prediction error is necessary, it is not sufficient: models must also respect physical constraints (e.g., thermodynamic consistency) and maintain interpretability and simplicity to avoid overfitting and to generalize reliably to new chemical spaces [21].

This led to the development of a multi-objective loss function that balances empirical accuracy, structural consistency, and physical feasibility. Here, for the first time, by integrating a multi-objective loss function that incorporated physics-based constraints, we were able to ensure that the model’s entropy predictions adhered to thermodynamic principles while also improving the model’s accuracy. Once we validated that the multi-objective loss function successfully enhanced entropy predictions, we extended this framework to predict the ΔΔG values. This allowed us to apply a similar approach to both entropy and binding free energy predictions. In the remainder of this paper, we first introduce the dataset and its properties, and the three key components of our novel multi-objective loss function: empirical accuracy, physical consistency, and model simplicity, detailing how each term is defined and incorporated into our hybrid modeling framework. We then describe the methodology for implementing this multi-objective approach within the context of host–guest systems, initially focusing on entropy prediction before extending the framework to binding free energy calculations.

## 2 MATERIALS AND METHODS

### 2.1 Dataset

Host-guest systems and Statistical Assessment of the Modeling of Proteins and Ligands (SAMPL) challenge benchmarks [34] introduce chemical hosts (size ~200–300 atoms) with pockets that enable strong binding to the corresponding compounds, called guests. Hosts bind their guests (size ~50–100 atoms) via the same basic forces that proteins use to bind their ligands, so they can serve as simple test systems for computational models of noncovalent binding [28]. Moreover, their small size and, in many cases, their rigidity can make it feasible to sample all relevant conformations. In this study, we utilized two distinct datasets for predicting entropy and binding free energy. This section introduces the first dataset which was specifically employed for entropy calculations, consisting of experimental data from 65 host-guest systems sourced from various research projects, including the SAMPL challenge activities. The host molecules in these systems primarily include octa-acid (OA), cucurbituril (CBn), and cyclodextrin, while the guest molecules are typically small organic compounds. For each system, topology and coordinate files were used to run computational simulations, allowing us to calculate the TΔS values for the complex, host, and ligand, where T is the absolute temperature in K and its value is always approximately ~ 300K.

#### Structure Preparation.

To process the raw data, we first gathered the solvated PDB files for these host-guest systems, which contained about 200–500 atoms per system. Each structure has topology and coordinate files which were taken as is from the source repository. These files were then used as inputs to generate separate receptor, ligand, and dry complex topology files using the ante-MMPBSA.py script from AmberTools [7] so that they are unsolvated for the purpose of performing NMA. The starting structure of the host-guest complexes was built, parameterized using the General Amber Force Field (GAFF), neutralized with sodium or chloride counter-ions using Antechamber, and solvated using the TIP3P water model through LEaP [31]. The resulting input files were then minimized and heated from 0K to 300K. Using the ptraj tool, 100 snapshots (frames) of each equilibrated system were extracted from the MD trajectory for the complex, receptor, and ligand individually. This step was crucial to create suitable inputs for the subsequent NMA simulations.

### 2.2 Physics-based Methods: NMA Simulation

The ground truth for the entropy is derived using the thermodynamic equation:

(1) -TΔS=ΔG-ΔH

where, ΔG and ΔH are the binding free energy and enthalpy changes, respectively, and T is the absolute temperature in K. In addition to relying on ground truth entropy values derived from experimental thermodynamic data, we sought to independently simulate entropy values using Normal Mode Analysis (NMA). This allowed us to directly compare the entropy estimates obtained from computational simulations with those calculated from experimental measurements. By benchmarking our model’s predictions against both sources, we aimed to refine the model’s ability to predict entropy, leveraging discrepancies to enhance its accuracy and physical consistency in representing thermodynamic properties.

NMA is a computational technique employed to estimate the vibrational entropy of molecular systems by analyzing their harmonic oscillations around an energy-minimized structure. By approximating the potential energy surface as harmonic, NMA facilitates the calculation of vibrational frequencies, which are integral to determining entropy contributions in molecular interactions [19]. In host-guest binding studies, NMA decomposes the total entropy (TΔS) into translational, rotational, and vibrational modes using Hessian matrix diagonalization derived from molecular dynamics (MD) trajectories [6]. We developed our dataset of entropy values by performing NMA on the host, guest, and complex molecular systems to obtain TΔS for each system using AmberTools. This dataset was then used in training and testing the machine learning model to adhere to the physical and thermodynamic constraints. For each snapshot, NMA calculates the translational, rotational, and vibrational entropy components for the complex, receptor, and ligand. These are then averaged over all frames. The total binding entropy change (ΔS) is obtained by combining the entropy values for the complex, receptor, and ligand.

The output includes the mean and standard deviation for each entropy component and the overall binding entropy, typically reported in kcal/mol at a specified temperature (300 K). By integrating entropy predictions into our multi-objective loss function, we have enhanced the predictive accuracy of the PG-RGCN model for binding free energy calculations. This hybrid approach allows us to explicitly account for the thermodynamic cycle, ensuring that entropy, along with enthalpy and free energy, is accurately represented in our model’s predictions.

### 2.3 Physics-based Methods : MM/PB(GB)SA for End-point Free Energy Calculations

The Molecular Mechanics Poisson-Boltzmann/ Generalized Born Surface Area (MM/PB(GB)SA) methods are widely used in computational chemistry to estimate the binding free energy (ΔΔG) of protein-ligand complexes [30]. These methods provide a way to decompose the free energy into individual components, including solvation and molecular mechanics terms [24, 26]. Specifically, MM/PBSA uses the Poisson–Boltzmann (PB) or generalized Born (GB) implicit solvent models to estimate solvation energies $\left(\Delta G_{\text{solv}}\right)$, while the molecular mechanics (MM) component is based on a classical force field [36]. The process begins by solvating the initial structure of the complex, followed by running a molecular dynamics (MD) simulation to generate a series of snapshots. These snapshots are then used to calculate the binding free energy. Typically, over 100 uncorrelated snapshots are extracted from the MD simulation to represent the structural ensemble. The solvation and molecular mechanics terms are calculated for each snapshot, and the average ΔΔG is determined by averaging over all the snapshots [16]. This approach allows for an efficient and robust estimation of binding free energy, which is crucial for understanding molecular interactions in drug design. The overall workflow is depicted in the figure below, which outlines the key steps in the single-trajectory MM/PBSA protocol. This method, implemented using AmberTools, provides a comprehensive framework for calculating binding free energies by integrating both molecular mechanics and implicit solvation models. Although they offer a practical balance between accuracy and speed, MM/PB(GB)SA approaches are also sensitive to parameter choices and system-specific assumptions, which can introduce systematic errors in the resulting estimates [8].

In this study, we utilized the single-trajectory MM/PB(GB)SA approach, which offers greater computational efficiency and reduced noise compared to the separate-trajectory method. This approach calculates the binding free energy change (ΔΔG) by analyzing a single molecular dynamics (MD) trajectory of the entire complex, thereby eliminating the need to generate separate trajectories for the receptor, ligand, and complex individually. The initial complex structures were solvated using an explicit water model. Subsequently, the solvated complexes underwent energy minimization with a maximum of 1000 minimization cycles, followed by a 50 ps heating phase from 1 K to 300 K under constant volume conditions. This was succeeded by 50 ps of density equilibration at 300 K and 1 bar pressure, and then an additional 2 ns of equilibration at constant pressure (1 bar) and temperature (300 K). Throughout these equilibration stages, atomic positions were restrained with a harmonic force constant of 2 kcal/mol/A^2^. All simulations, including the production runs, were performed using the GPU-accelerated pmemd.cuda engine, employing Langevin dynamics with a collision frequency of 2 ps^−1^ and a 2 fs integration time step. The SHAKE algorithm was applied to constrain bonds involving hydrogen atoms. Electrostatics were treated using the Particle Mesh Ewald (PME) method with a non-bonded cutoff distance of 9 Å. Trajectory coordinates were saved every 10 ps [16].

Following these preparations, MD simulations were conducted, from which a substantial number of snapshots were extracted. After stripping solvent molecules, the average binding free energy calculated over these snapshots was assigned as the system’s ΔΔG. MM/PB(GB)SA also provides the mean and standard deviation for each energetic component contributing to ΔΔG. This methodology relies on the assumption that binding does not induce significant conformational changes, making it particularly well-suited for the host–guest systems examined in this work.

### 2.4 Deep Learning Method: Physics-guided Rule-based Graph Convolutional Network

The model architecture is based on Graph Convolutional Networks (GCNs), which are used to capture the structural relationships between atoms in the molecule [37]. Each atom is treated as a node, and each bond is treated as an edge in the graph. The GCN learns to aggregate information from neighboring atoms through multiple graph convolution layers. The model employed in this study is an extension of the Physics-Guided Rule-based Graph Convolutional Network (PG-RGCN), originally designed to predict the entropy term in binding free energy calculations [31]. This section elaborates on the approach followed to integrate both structure-based and physics-based features, providing a robust framework for learning accurate molecular predictions that respect the underlying physics of molecular interactions for binding free energy calculations. The dataset was obtained from the Mobley’s benchmark sets, consisting of 72 host-guest systems where 15 physics-based features were considered, which were computed using the GBNSR6 model available within the AmberTools module, as outlined in [5]. To ensure the robustness and generalizability of our deep learning models, we applied a 4-fold cross-validation approach, splitting the host-guest dataset for both entropy and binding free energy predictions. This division allowed us to train and test our models on substantial portions of the data, reserving a significant subset for unbiased model evaluation. This cross-validation approach was critical in validating the predictive accuracy and transferability of our models to real-world applications, ensuring that the trained models could generalize well to unseen data.

#### Structure-based Features.

Structure-based features are extracted using a deep chem featurizer, which calculates atomic properties such as atom type, hybridization state, valence, and aromaticity [5]. These features are then encoded as one-hot vectors and combined to form a feature matrix for each molecule. The feature matrix is structured as N×M, where N is the number of atoms and M is the number of features per atom. This matrix serves as the input for the subsequent graph convolutional network.

#### Physics-based Features.

The physics-based features, P, of each molecule include energy terms like electrostatic energy (EELEC), Van der Waals energy (VDWAALS), solvation energy, and 1–4 electrostatic energy (1–4-eel), which are calculated using the GBNSR6 model in AmberTools 2020 [6]. These features are concatenated with the structure-based features to provide a comprehensive input representation for the model. In short, GBNSR6 model is executed on the complex, host, and guest PDB files. Electrostatic energy (EELEC), non-polar solvation energy (ESURF) and polar solvation energy (EGB) are extracted accordingly [15, 17]. The PBSA model is employed to calculate Van der Waals (VDWAALS) energy separately as it is required for total energy calculations. The list of physics-based features is presented in Table 1.

### 2.5 Proposed Multi-Objective Loss Function

In recent years, the use of multi-objective loss functions has gained significant attention in machine learning, particularly for improving model performance by incorporating domain-specific knowledge [13]. Multi-objective optimization optimizes multiple objectives simultaneously, each representing a different aspect of model behavior, rather than focusing on a single objective [22]. A key contribution to this field is the introduction of Physics-Guided Neural Networks (PGNN) in lake temperature modeling, where traditional machine learning loss functions were combined with physics-based terms to enforce physical constraints [11]. This approach demonstrated that multi-objective loss functions could improve model generalizability and interpretability by guiding optimization towards solutions that respect both data-driven and domain-specific requirements.

To train our model, we introduce a novel multi-objective loss function that combines empirical loss with structural error and physical inconsistency terms. This combined loss ensures that the model not only fits the data well but also adheres to physical principles that govern molecular interactions. The multi-objective loss function is defined as:

(2) $$\text{Multi-Objective Loss}=\text{L}\hat{\text{o}}\text{ss}(Y,\overset{ˆ}{Y})+\|w{\|}_{2}^{2}+\lambda_{\text{PHY}}{\text{L}\hat{\text{o}}\text{ss}}_{\text{PHY}}(\overset{ˆ}{Y})$$

Where: - $\text{L}\hat{\text{o}}\text{ss}(Y,\overset{ˆ}{Y})$ is the Empirical Loss (Root Mean Squared Error between the predicted and actual values), - $\|w{\|}_{2}^{2}$ is the Structural Loss (L2 regularization), - ${\text{L}\hat{\text{o}}\text{ss}}_{\text{PHY}}(\overset{ˆ}{Y})$ is the Physical Inconsistency Loss (penalizing violations of physical laws).

#### 2.5.1 Empirical Loss Term.

The first and foremost goal of the model is to minimize the discrepancy between predicted and observed data. This is typically measured using Root Mean Squared Error (RMSE), which quantifies how well the model fits the experimental data [23]. By minimizing this term, the model strives to provide predictions that are as close as possible to the true values of calculations.

(3) $$\text{RMSE}=\sqrt{\frac{1}{N}\sum_{i=1}^{N}{\left(y_{\text{pred},i}-y_{\text{true},i}\right)}^{2}}$$

where $y_{\text{pred},i}$ and $y_{\text{true},i}$ are the predicted and true (experimental) values for binding energy and entropy for complex i, respectively, and N is the number of samples.

#### 2.5.2 Structural Loss Term.

The second component of the loss function addresses the *complexity* of the model itself. While increasing the complexity of the model can improve its fit to the training data, it may also lead to *overfitting*, where the model captures noise rather than meaningful patterns. Overfitting is particularly detrimental in predictive tasks such as free energy calculations, where generalizability to unseen data is crucial [29]. To mitigate this, a ‘structural penalty’ is introduced, which is implemented implicitly through the use of L2 regularization and dropout layers within the model. These components serve to penalize overly complex models and prevent overfitting by regularizing the weights of the neural network layers [14]. In our model, L2 regularization is applied to all dense layers, such as:

(4) $$\text{Dense Layer Regularization}=\|w{\|}_{2}^{2}$$

where w represents the weights of the layer. This regularization term encourages the model to keep the weight values small, ensuring that the learned model does not overly rely on any particular feature or data point. Dropout layers are also introduced after each dense layer to further prevent overfitting by randomly setting a fraction of input units to zero during training. The MaxNorm constraint further ensures that the magnitude of the weights is bounded, preventing the model from learning unnecessarily complex features that don’t generalize well. By controlling the model’s complexity, this term ensures that the model maintains a balance between fitting the data well and not overfitting, allowing it to generalize effectively to new, unseen data. The structural loss term has been critical in improving the model’s performance. Adding this term consistently resulted in better training and testing losses, as well as a lower mean absolute difference between the predicted and true values of binding free energy. This demonstrates the importance of incorporating structural constraints into the model, as it improves generalization and prevents the model from learning spurious correlations.

#### 2.5.3 Physical Inconsistency Loss Term.

While empirical accuracy is crucial, it does not guarantee that the predictions align with fundamental physical principles. In molecular systems, one such principle is the additivity of binding energies [26], where the binding free energy of a complex $\left(\Delta G_{\text{complex}}\right)$ is related to the sum of the energies of the host and guest molecules:

(5) $$\Delta \Delta G_{\text{pred}}=\Delta G_{\text{complex}}-\left(\Delta G_{\text{host}}+\Delta G_{\text{guest}}\right)$$

The physical inconsistency loss ensures that the model predictions respect the underlying physics of molecular interactions, particularly the thermodynamics of host-guest binding. This term is derived from the difference between the predicted binding free energy $\left(\Delta \Delta G_{\text{pred}}\right)$ and the energy differences computed from physical features (i.e., electrostatic, solvation, and Van der Waals energies).

Mathematically, the physical inconsistency loss is computed as:

(6) $${\text{L}\hat{\text{o}}\text{ss}}_{\text{PHY}}=\sqrt{\frac{1}{n}\sum_{i=1}^{n}{\left({\overset{ˆ}{Y}}_{i}-\Delta G_{\text{physics},i}\right)}^{2}}$$

Where ${\overset{ˆ}{Y}}_{i}$ is the predicted binding free energy for the i-th sample and $\Delta G_{\text{physics},i}$ is the energy difference predicted using the MM/PB(GB)SA results. Similarly, for entropy calculations, this term would be calculated by taking the difference between the predicted values and the NMA results.

The physical inconsistency loss directly penalizes any predictions that deviate from the expected thermodynamic relationships. By minimizing this term during training, the model is encouraged to make predictions that are not only statistically accurate but also physically plausible. This ensures that the model learns to respect the underlying thermodynamic principles governing binding interactions. In practice, the physical inconsistency loss term is scaled by a hyperparameter $\lambda_{\text{PHY}}$, which controls the relative weight of the physical consistency term in the total loss function. This parameter allows for tuning the balance between empirical accuracy and physical consistency, ensuring the model is both precise and physically realistic.

**Figure 4:**
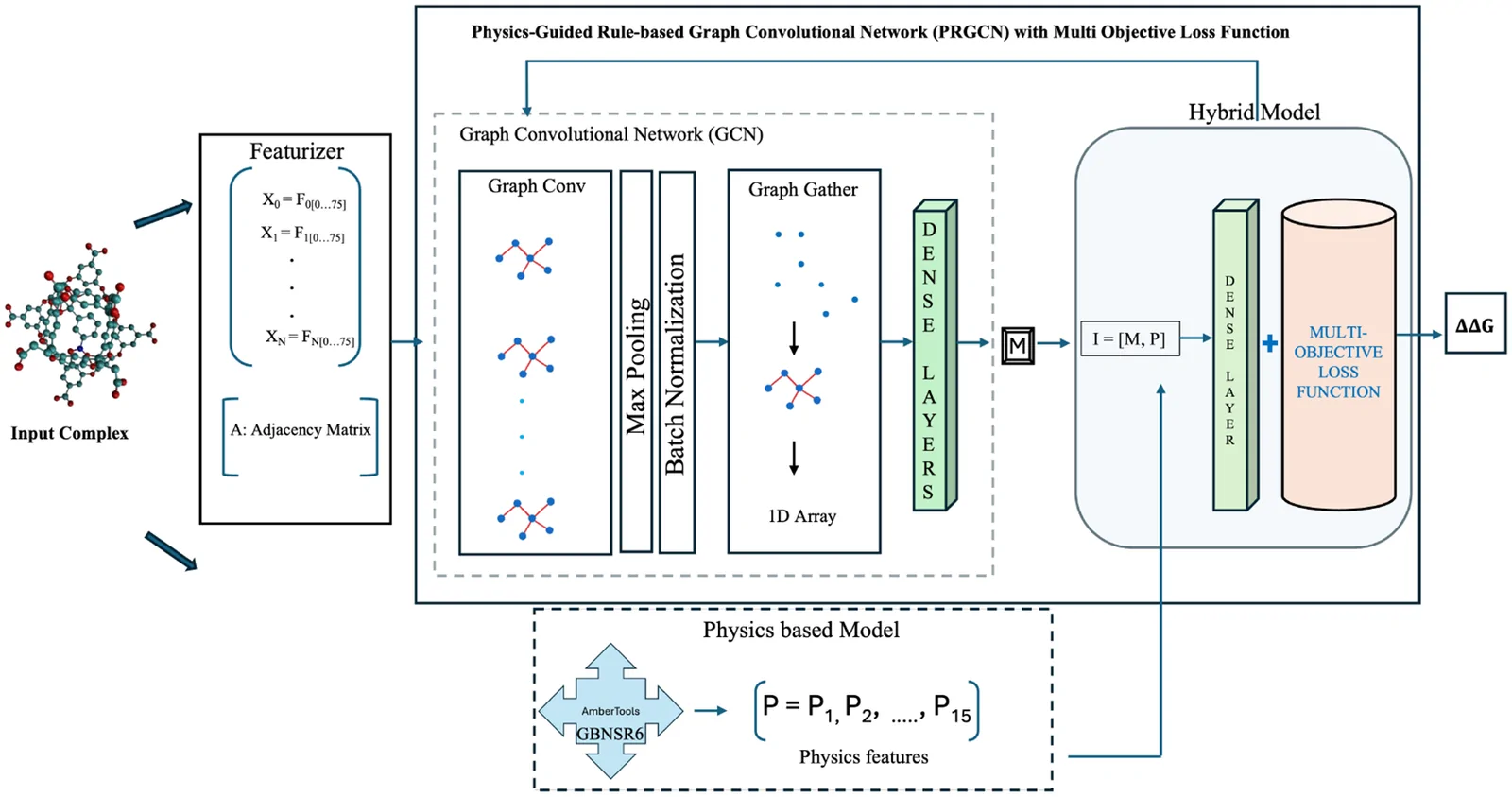
The PG-RGCN model processes a sample complex (octa-acid and 4-Bromoadamantane-1-carboxylic acid) using a Graph Convolutional Network (GCN) and Implicit Solvent Model. The model calculates physical measures for the protein, ligand, and complex, storing the output in vector P. This is concatenated with the neural network output M and fed into the Hybrid PG-RGCN model, which trains to minimize the loss function. The final output, ΔΔG, is reported after convergence.

By integrating all these components, we significantly enhanced the predictive accuracy of the PG-RGCN model. The multi-objective loss function was first evaluated in the context of entropy prediction, where it consistently outperformed traditional RMSE-based approaches. Specifically, when applied to the prediction of ΔS (entropy changes) in host–guest systems, our approach yielded superior performance, demonstrating both improved accuracy and greater alignment with underlying thermodynamic principles. Encouraged by the improved results from entropy prediction, we extended this approach to predict the binding free energy (ΔΔG) and applied the same multi-objective loss function to this more complex problem. The model architecture can be seen in Figure 4.

## 3 RESULTS AND DISCUSSION

### 3.1 Multi-Objective Loss Function for Entropy Calculations

**Figure 5:**
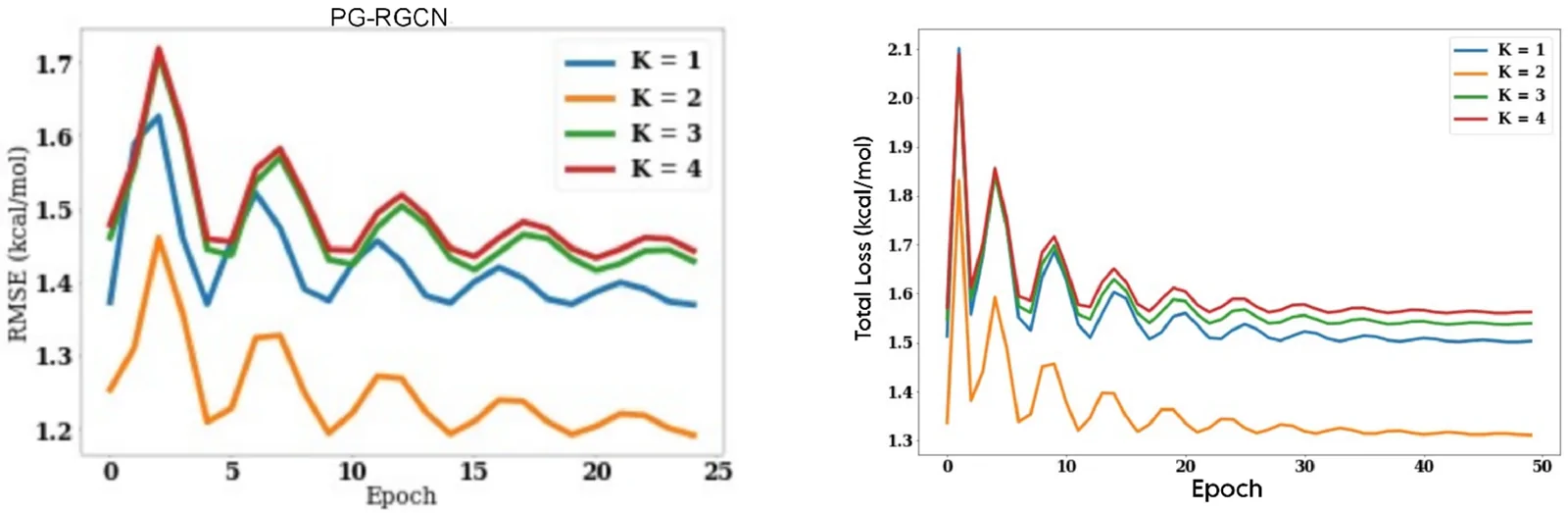
Comparison of 4-fold cross-validation results for entropy prediction: (Left) without multi-objective loss function, (Right) with multi-objective loss function.

**Figure 6:**
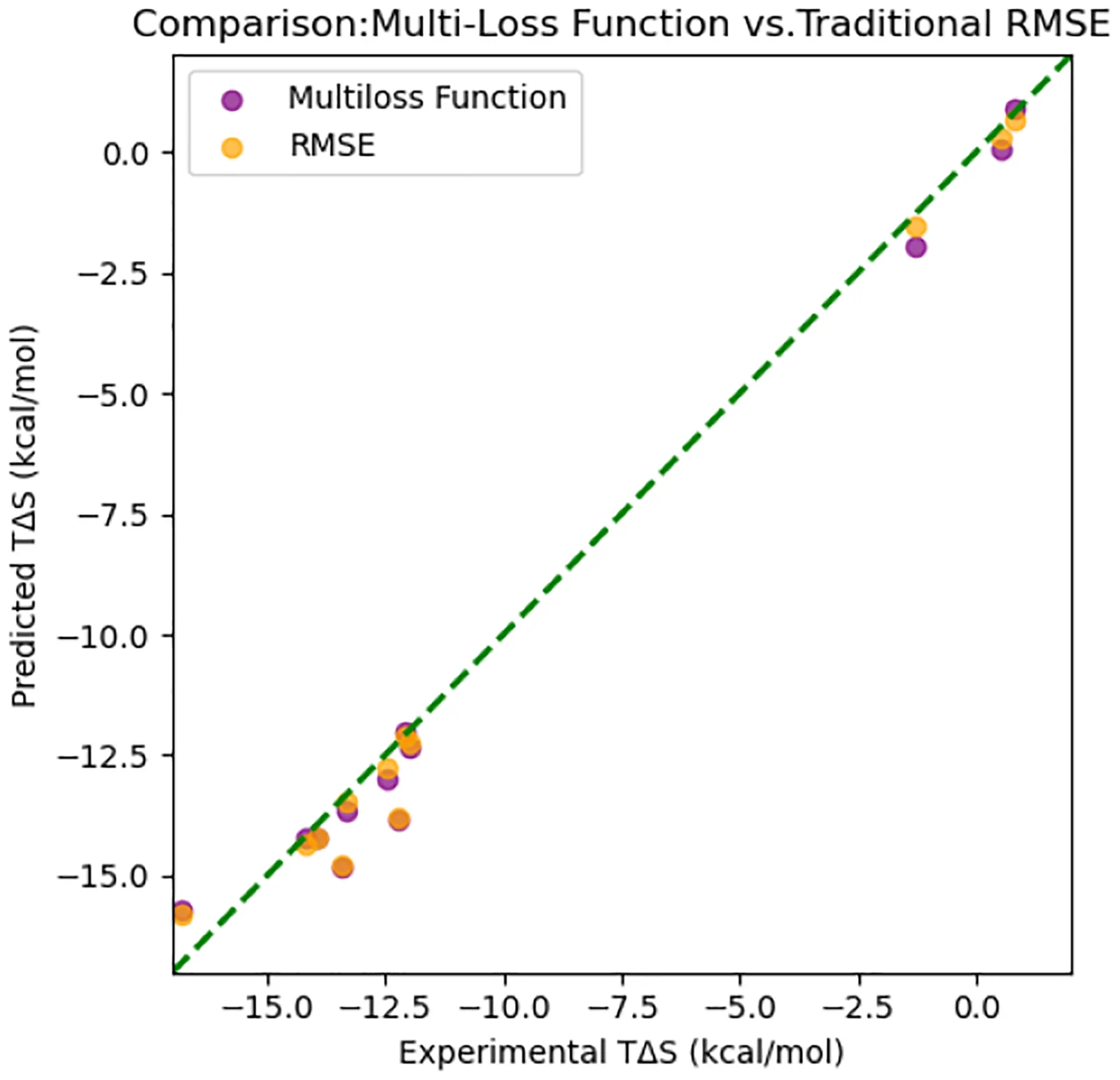
The plot depicts the comparison between Experimental and Predicted TΔS values (in kcal/mol) with and without incorporating multi-objective loss terms in the PG-RGCN model.

We obtained entropy values for the complex, receptor, and ligand systems from NMA calculations, including vibrational, translational, and rotational entropy as well as the total entropy (TΔS) values reported in kcal/mol for each component. These values were utilized as physically motivated references to assess the physical consistency of our model’s entropy predictions. Specifically, the total TΔS values derived from Normal Mode Analysis (NMA) simulations provided a benchmark for evaluating whether the predicted entropy changes adhered to established thermodynamic principles. We used the introduced dataset of 65 host-guest systems to train a machine learning model for entropy prediction. We began by evaluating the performance of the model using K-fold cross-validation, where K=4, for predicting the entropy change (TΔS) of the host-guest systems. The results of this cross-validation are depicted in Table 2 and the visualization of the loss across various folds can be observed from graphs shown in Figure 5. These graphs highlight the performance of the model in predicting entropy based on the NMA-derived entropy values. In Figure 5, the subplot on the left shows the 4-fold cross-validation results for entropy prediction without the multi-objective loss function. As seen in the graph, the total loss fluctuates across different epochs, indicating some instability during the early stages of training. This fluctuation suggests that the model is struggling to find a stable solution and is prone to overfitting, especially in the early epochs of training. The high variance observed is typical of models that lack additional regularization techniques, such as structural loss or physical consistency terms. In contrast, the subplot on the right shows the 4-fold cross-validation results for entropy prediction using the multi-objective loss function. The introduction of the multi-objective loss function has significantly improved the stability of the training process. The graph exhibits a much smoother total loss curve with fewer fluctuations, which indicates that the model has converged more quickly and efficiently. The reduction in fluctuations suggests that the model is better able to generalize, with a more stable convergence, and is less prone to overfitting. The successful prediction of entropy values using the multi-objective loss function laid the groundwork for extending the model to predict binding free energy. After training and testing the model on an 80:20 train-test data split, the results showed that, before implementing the loss function, the model had a train loss of 1.35 kcal/mol, a test loss of 2.04 kcal/mol, and a MAD of 5.80 kcal/mol. Following the integration of the loss function, the model demonstrated modest improvements in the training and test losses, with train loss dropping to 1.30 kcal/mol and test loss rising slightly to 2.10 kcal/mol. While the test loss remained relatively unchanged, the MAD showed a marked improvement, decreasing dramatically from 5.80 kcal/mol to 1.07 kcal/mol. This significant reduction highlights the enhanced accuracy of the model’s predictions, with a substantial improvement in the proximity of predicted values to the true values, even as the test loss showed minimal change. The inclusion of entropy as a feature, coupled with the multi-objective loss function, led to more reliable and precise results, as illustrated in Figure 6. These findings not only demonstrate the model’s improved performance but also provided valuable insights that guided its refinement for more complex predictions, such as binding free energy. Building on these promising results, the model was subsequently adapted to predict ΔΔG, focusing on the physics features relevant to binding energy rather than entropy.

### 3.2 Multi-Objective Loss Function for Binding Free Energy Calculations

**Figure 7:**
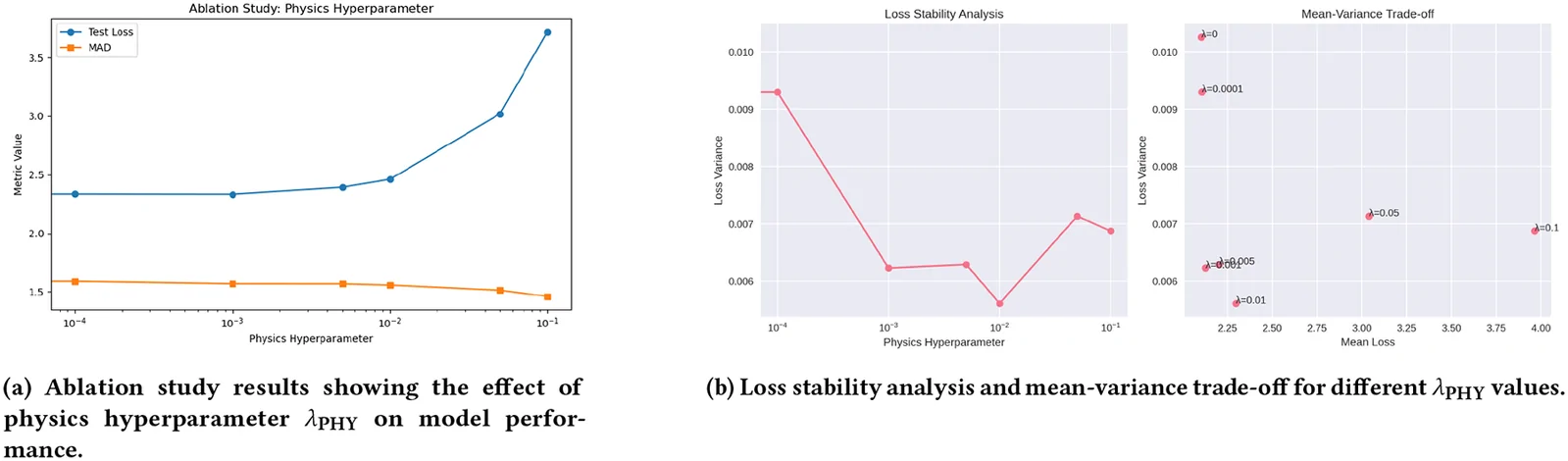
Complementary analyses of $\lambda_{\text{PHY}}$: (a) Ablation study results and (b) stability and trade-off analysis.

The model was trained using the Adam optimizer with a dynamic learning rate schedule, where the learning rate decays exponentially. This allows the model to smoothly converge towards the optimal solution. Figure 7a shows the ablation study conducted to select an appropriate physics hyperparameter. The plot demonstrates the trade-off between test loss (blue) and MAD (orange), across different hyperparameter values. The stable performance region (10^−4^ to 10^−2^) suggests an optimal $\lambda_{\text{PHY}}$ value around 0.005, balancing model accuracy with physical consistency. Early stopping was employed during training, halting the process when the validation loss ceases to improve, thereby preventing overfitting and ensuring better generalization on unseen data. Furthermore, additional studies, such as loss stability analysis and convergence analysis, helped the process of choosing optimal hyperparameters that improved the model’s performance and training. The figure 7b presents two complementary analyses of model stability. The left plot shows that loss variance decreases with increasing $\lambda_{\text{PHY}}$ up to 0.01, reaching maximum stability before slightly rising. The right plot illustrates the mean-variance trade-off, where low $\lambda_{\text{PHY}}$ values (10^−4^, 10^−3^) yield higher variance despite lower mean loss, and higher values (0.05, 0.1) lead to increased mean loss. The mid-range $\left(\lambda_{\text{PHY}}=0.005-0.01\right)$ provides the best balance, with moderate mean loss (2.75–3.0) and low variance (~ 0.006), indicating an optimal stability trade-off.

The total loss, which is a combination of empirical loss, structural loss, and physical consistency loss, exhibits a sharp decline early in the training process. The model shows significant learning within the first few epochs, indicating rapid convergence towards an optimal set of weights. However, after the initial drop, the loss fluctuates, reflecting some minor adjustments made by the optimizer. These oscillations are typical when using a dynamic learning rate, which is designed to allow fine-tuning of the model’s predictions as it nears convergence.

**Figure 8:**
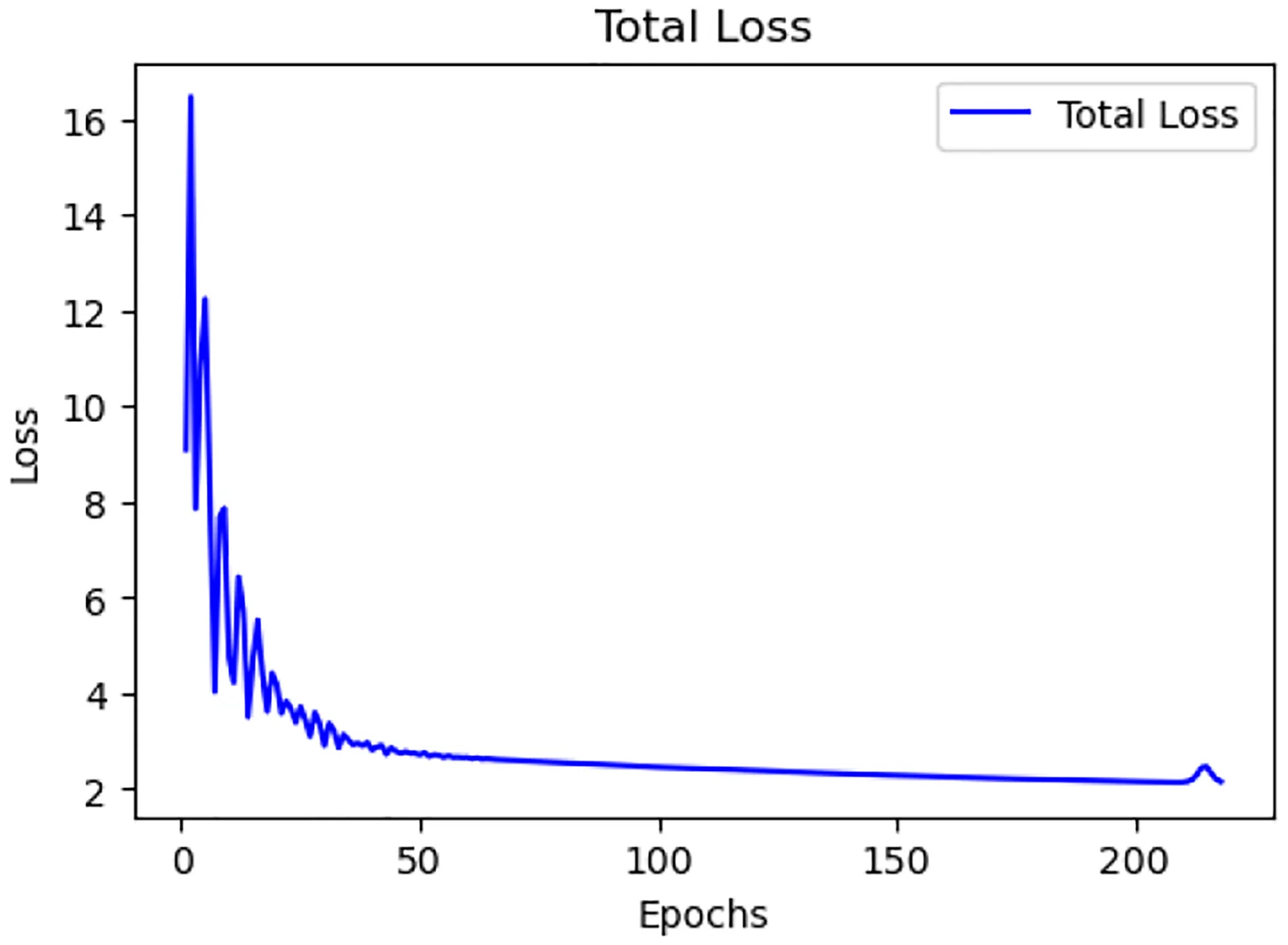
Total loss vs Epochs during model training. Total loss calculated for 500 epochs to predict binding free energy. The loss stabilizes and converges towards a minimum value at around 218 epochs, with a train loss of 2.16 kcal/mol.

As shown in the graph (Figure 8), the total loss decreases steadily, reaching a plateau as the model continues training. The final training loss of 2.16 kcal/mol, as indicated in the training logs, further confirms that the model has stabilized. The early stopping mechanism ensures that the model does not overfit to the data, and the test loss of 2.34 kcal/mol, with a MAD of 1.54 kcal/mol, demonstrates that the model is able to generalize effectively to unseen host-guest systems. This pattern validates the training strategy, with the combination of dynamic learning rate decay and early stopping ensuring optimal model performance without overfitting.

To evaluate the model’s performance, we first conducted K-fold cross-validation with K=4. This approach allowed us to test the model’s generalization ability and its robustness across different splits of the data. The results from the 4-fold cross-validation (Table show a clear improvement when the multi-objective loss function is used. The mean test loss of the multi-loss model (2.48±0.74) kcal/mol is significantly lower compared to the model without the multi-loss function (3.51±0.87) kcal/mol. Additionally, the MAD for the multi-loss model is 1.65±0.49 kcal/mol, indicating that the model with the multi-objective loss function makes predictions with much smaller deviations from the true values. The model without the multi-objective loss function, on the other hand, has a higher MAD, showing that it struggles with larger prediction errors. This demonstrates that the inclusion of the multi-objective loss function improves the model’s ability to generalize and make accurate predictions.

Following the validation using 4-fold cross-validation, we analyzed the impact of different loss terms—traditional RMSE, structural error, and multi-objective loss function—on the predictions. From Table 5, it is evident that the model with the multi-loss function exhibits significantly better performance in predicting binding free energy. The multi-loss model achieves a training loss of 2.15 kcal/mol and a test loss of 2.34 kcal/mol. The MAD for this model is 1.54 kcal/mol, indicating the lowest deviation between the predicted and experimental values. This demonstrates that the multi-objective loss function, which incorporates empirical accuracy, structural error, and physical consistency, enables the model to effectively learn and generalize from the data.

In comparison, the pure RMSE model performs less effectively, with a higher training loss of 2.97 kcal/mol and a test loss of 3.41 kcal/mol. The MAD of 2.83 kcal/mol further suggests that this model struggles to make accurate predictions, showing a greater discrepancy between predicted and experimental binding free energies. The model that incorporates only the structural loss function shows some improvement over the pure RMSE model. With a training loss of 2.10 kcal/mol and a test loss of 2.27 kcal/mol, the model demonstrates better learning, but it still falls short compared to the multi-loss function model. The MAD of 2.49 kcal/mol indicates that while the structural loss function improves performance, it is not as effective as the multi-objective loss function. The significant improvement in mean absolute difference suggests that the multi-loss approach is especially effective at reducing large prediction errors, which is crucial for practical applications where outliers could lead to poor decision-making.

**Figure 9:**
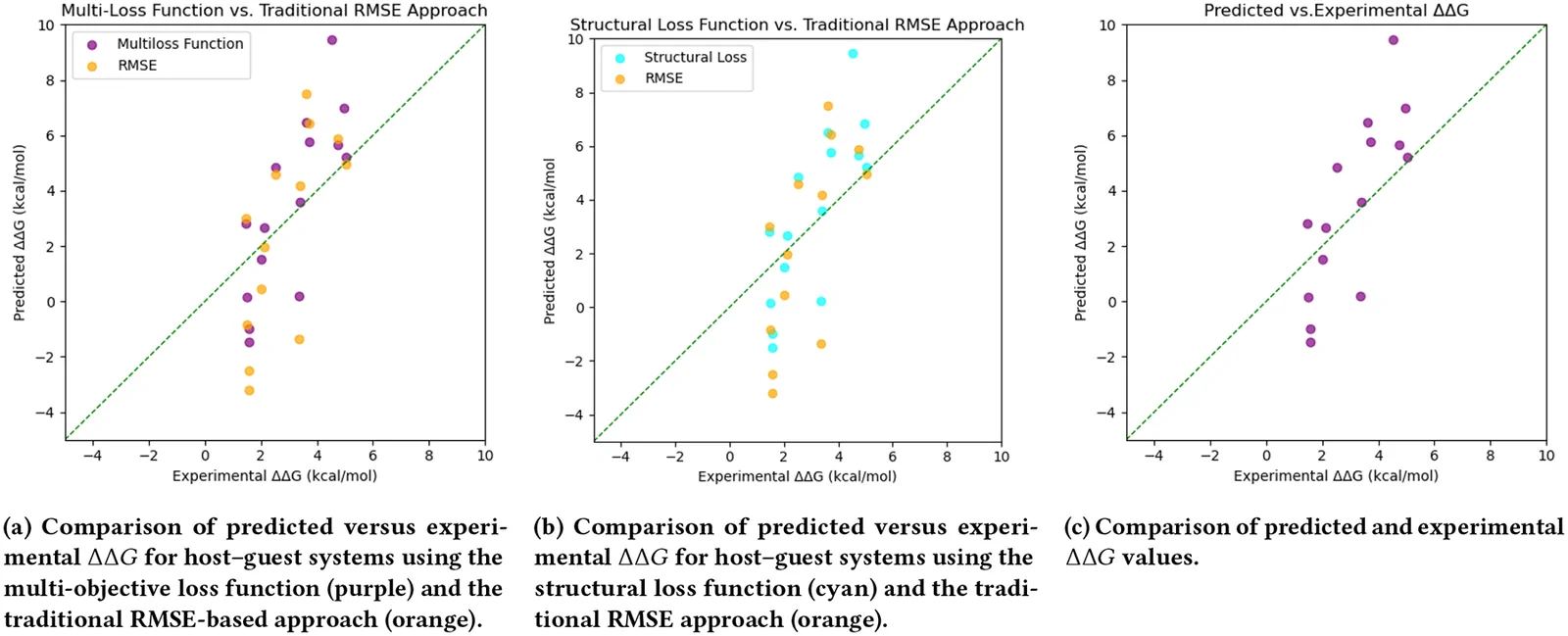
Comparison of ΔΔG prediction with the physics-guided rule-based graph convolutional (PG-RGCN) model. Each point represents a single system, and the dashed green line indicates perfect agreement between predicted and experimental values.

The scatter plots shown in Figure 9 further confirm these findings. The multi-loss function model shows a strong diagonal correlation between the predicted and experimental binding free energy values, indicating more accurate predictions. The RMSE model’s scatter plot, on the other hand, shows greater dispersion, reflecting its inability to consistently predict binding free energies. The scatter plot for the structural loss function model shows some improvement over the RMSE model but still lacks the level of precision seen in the multi-loss function approach.

The results of K-fold cross-validation, combined with the table comparisons, demonstrate the value of the multi-objective loss function in improving the predictive accuracy and generalization of the model. The multi-loss approach has not only achieved a lower test loss (about 31.5% improvement), but more significantly, it has reduced the mean absolute difference between predicted and true values by approximately 45.8%. This is a substantial improvement in the model’s predictive accuracy.

However, as observed when comparing the model’s performance in predicting both entropy and binding free energy, the benefits of physics-informed approaches can vary significantly depending on the dataset. For some problems, such as binding free energy calculations, physics constraints are essential for ensuring convergence. In contrast, for other tasks like entropy predictions, the benefits of incorporating physical consistency may be more subtle or even redundant if the data already strongly reflects the underlying physics. That said, the physics inconsistency component may still provide benefits that are not immediately apparent in the training loss curves alone, underscoring its potential value in more complex or less well-characterized molecular systems.

## 4 CONCLUSION

In this paper, we presented a novel approach to predicting protein-ligand binding free energies using a multi-objective loss function integrated into a Physics-guided Rule-based Graph Convolutional Network. The model combines empirical loss, physical consistency loss, and structural complexity penalties to ensure that the predictions not only fit the available data but also adhere to fundamental thermodynamic principles. We first demonstrated the model’s effectiveness by predicting entropy values using Normal Mode Analysis (NMA) data, a significant step forward given the computational challenges of entropy calculations for large molecular systems. Traditionally, entropy calculations have been computationally expensive and challenging, particularly for large molecular systems. With AmberTools for entropy calculation, we efficiently computed entropy values for host-guest systems, bridging the gap between high-quality physical simulations and data-driven machine learning. When applied to binding free energy prediction, the model showed promising results, especially with physical consistency constraints. This approach yielded more accurate, physically meaningful results compared to traditional black-box methods. A key finding is that, while both models had similar loss values, the physics-informed model significantly reduced absolute error, suggesting that traditional models may miss important physical considerations. Future research should focus on scaling the model to larger datasets, such as PDBbind [33], to assess its scalability, although it would require significantly more computational resources. In addition, we plan to optimize the multi-objective loss function further using algorithms like PCGrad [35] to address the conflict between the different losses. Both the dataset and source code of our model implementation can be found publicly available at our GitHub repository - https://github.com/COMB-Lab/Multi-Objective-Loss-Function-for-Free-Energy-Calculations

## Figures and Tables

**Figure 2:**
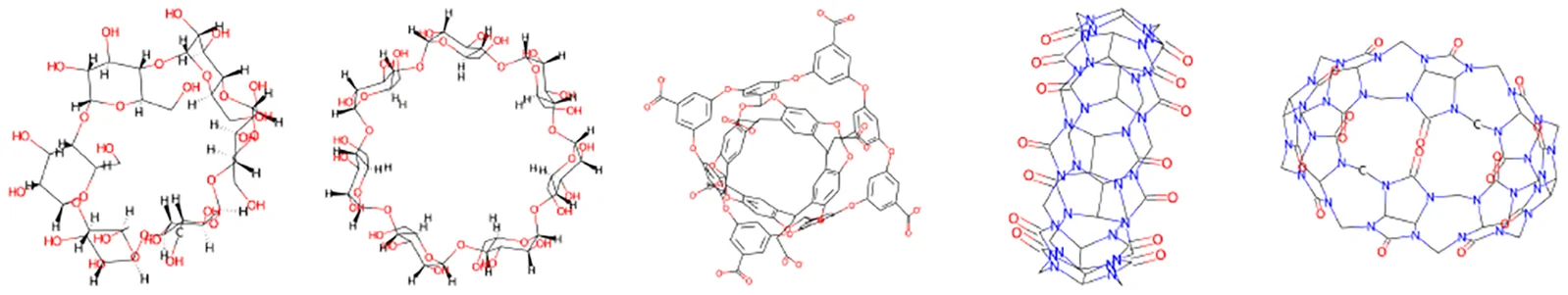
SAMPL hosts used in this research (from left to right): α-cyclodextrin, β-cyclodextrin, OA, CB7, CB8 [34].

**Figure 3:**
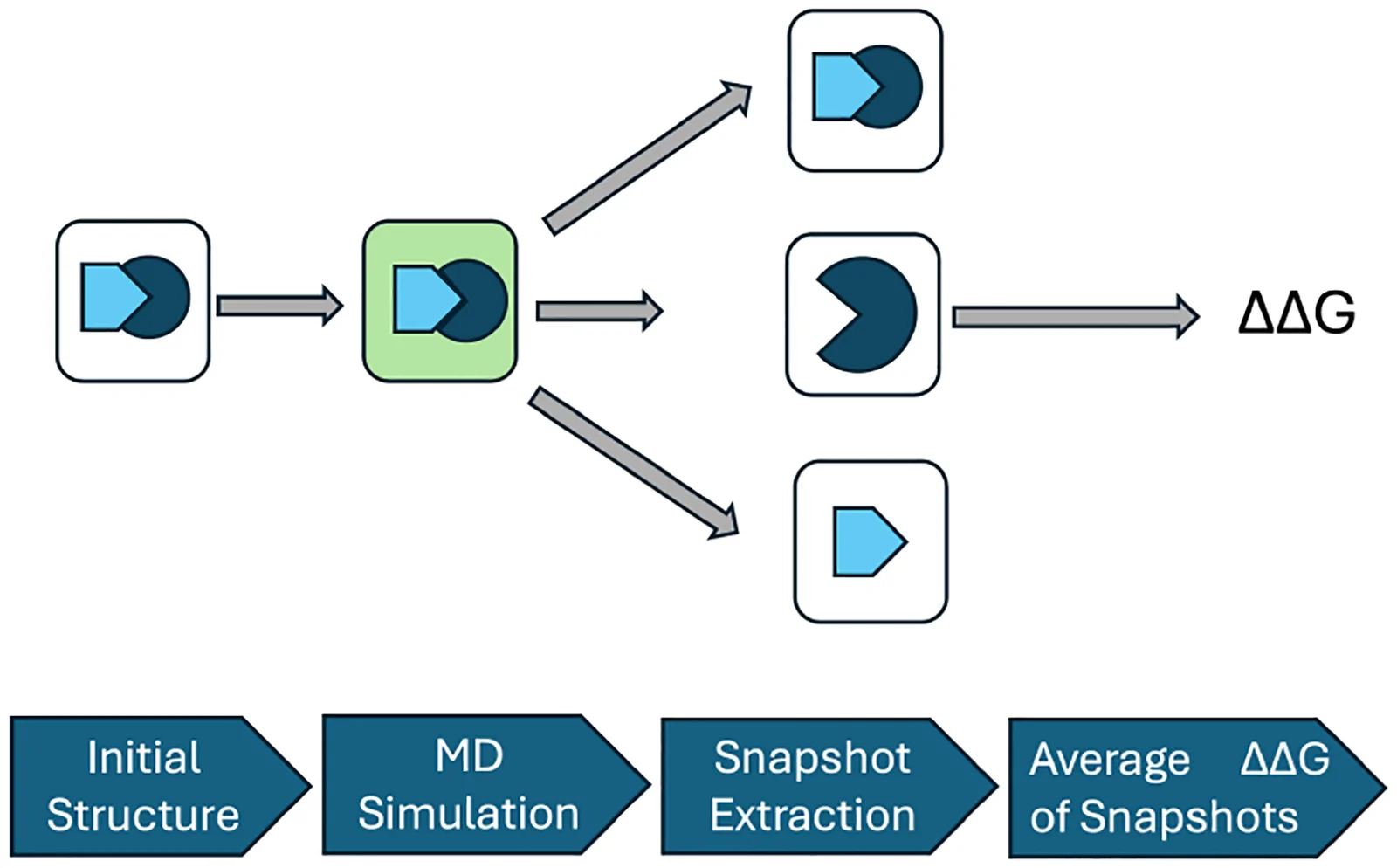
MM/PBSA flowchart for calculating binding free energy. The process starts with the initial structure of the complex, followed by MD simulation, snapshot extraction, and average binding free energy calculation [16].

**Table 1:** Physics-Based Features Calculated for Complex, Host, and Guest Structures using GBNSR6 and PBSA. See [4] for details.

Parameter	Description	Count
Etot	Total Energy	3
VDWAALS	Van der Waals Energy	3
EELEC	Electrostatic Energy	3
ESURF	Non-polar Solvation Energy	3
EGB	Polar Solvation Energy	3
**Total**		**15**

**Table 2:** Model performance for predicting entropy change (TΔS) of the host-guest systems using 4-fold cross-validation. All values are in kcal/mol.

Model	Train Loss	Test Loss
Without Loss Function	1.56±0.10	1.59±0.29
With Loss Function	1.44±0.10	1.47±0.28

**Table 3:** Train Loss, Test Loss, and Mean Absolute Difference in kcal/mol for ΔΔG predictions.

Metric	Value (kcal/mol)
Train Loss	2.16
Test Loss	2.34
Mean Absolute Difference	1.54

**Table 4:** K-fold cross-validation results for binding free energy (ΔΔG) prediction (kcal/mol), where K=4. The table highlights the improved performance of the multi-objective loss function compared to the model that does not use the multi-objective loss function.

Model Type	Mean Train Loss	Mean Test Loss	Mean Absolute Difference
ΔΔG with Multi-loss	2.14±0.18	2.48±0.74	1.65±0.49
ΔΔG without Multi-loss	3.08±0.29	3.51±0.87	3.14±0.79

**Table 5:** Error in calculating binding free energy ΔΔG. Values of loss function comparisons are in kcal/mol.

Prediction Type for ΔΔG	Train Loss	Test Loss	Mean Absolute Difference
With RMSE	2.97	3.41	2.83
With Structural Loss term	2.10	2.27	2.49
With Multi-Objective Loss Function	2.15	2.34	1.54

## CCS CONCEPTS

• Computing methodologies → Neural networks; Artificial intelligence; • Applied computing → Physics; Chemistry.
